# Types of second primary cancers influence survival in chronic lymphocytic and hairy cell leukemia patients

**DOI:** 10.1038/s41408-019-0201-0

**Published:** 2019-03-26

**Authors:** Guoqiao Zheng, Subhayan Chattopadhyay, Amit Sud, Kristina Sundquist, Jan Sundquist, Asta Försti, Richard S. Houlston, Akseli Hemminki, Kari Hemminki

**Affiliations:** 1 Division of Molecular Genetic Epidemiology, German Cancer Research Center (DKFZ), Im Neuenheimer Feld 580, D-69120 Heidelberg, Germany; 2 Faculty of Medicine, University of Heidelberg, Heidelberg, Germany; 3 Division of Genetics and Epidemiology, The Institute of Cancer Research, London, UK; 4 Center for Primary Health Care Research, Lund University, 205 02 Malmö, Sweden; 5 Department of Family Medicine and Community Health, Department of Population Health Science and Policy, Icahn School of Medicine at Mount Sinai, New York, USA; 6 Center for Community-based Healthcare Research and Education (CoHRE), Department of Functional Pathology, School of Medicine, Shimane University, Matsue, Japan; 7 Division of Molecular Pathology, The Institute of Cancer Research, London, UK; 8 Cancer Gene Therapy Group, Faculty of Medicine, University of Helsinki, Helsinki, Finland; 9 Comprehensive Cancer Center, Helsinki University Hospital, Helsinki, Finland

Second primary cancers (SPCs) are becoming more common as the survival in cancer is improving, and they are of main concern in cancers of good survival because they may cause early mortality. Here we want to test the hypothesis that the type of SPC is critical for survival and we further posit that the survival time can be predicted from the fatality of these cancers as first primary cancers. We test the hypotheses on two leukemias with good survival, the common chronic lymphocytic leukemia (CLL) and the rare hairy cell leukemia (HCL). In the comparison of survival rates we use relative survival to avoid biases in the definition of the cause of death.

CLL is characterized by the gradual accumulation of small phenotypically mature malignant B lymphocytes in the blood, bone marrow, and lymph nodes^1^. CLL may be preceded by monoclonal B-cell lymphocytosis, which evolves to CLL through genetic changes including somatic mutations and chromosomal aberrations^1^. Many patients are diagnosed at an asymptomatic stage and may not initially require treatment. Management of symptomatic patients includes chemotherapy with alkylating agents and purine analogs, combination of chemotherapy and immunotherapy, and drugs that target key signaling pathways^1,2^. Survival rates for patients with CLL have continuously improved mainly due to more efficient treatment^2,3^. Increased survival rates increase the likelihood of SPCs, which may potentially interfere with survival. Elevated risks for SPC have been reported in patients with CLL, including non-melanoma skin cancer, melanoma, sarcoma, and lung, renal, and prostate cancers^4,5^. It was reported that CLL patients with second malignancies have a worse relative survival than non-CLL patients with the same second malignancies^6,7^.

HCL is a B-cell disease with common somatic *BRAF* mutations. Many patients have an indolent course and no therapies are required^8^. Therapies were developed in 1990 based on purine analogs, which achieved good response rates, and more recently targeted treatments have become available including inhibition of the mutated BRAF kinase^8^. Since 1990, relative survival has been close to the background population among patients diagnosed before the age of 60 years and has now improved to ~90% even among elderly people^8^. Increased risks of SPCs in HCL patients have been reported for Hodgkin and non-Hodgkin lymphoma (NHL) and for thyroid cancer^9,10^.

We used data from the Swedish Cancer Registry to assess survival in CLL and HCL with and without SPCs. In addition, we grouped SPCs into three ‘prognostic groups’ based on 5-year relative survival of these cancers as first primary cancer^11,12^: ‘good survival’ (relative survival > 60%) included cancers in the lip, larynx, anus, breast, cervix, endometrium, prostate, testis, male genitals, kidney, bladder, melanoma, skin (squamous cell, SCC), eye, thyroid gland and endocrine, and Hodgkin lymphoma; ‘moderate survival’ (40–60%) included cancers in the remaining upper aerodigestive tract, salivary glands, small intestine, colorectum, female genitals, bone and connective tissue, and NHL and ‘poor survival’ (<40%) included cancers in the stomach, esophagus, liver, pancreas, lung, ovary and nervous system, and myeloma. Relative survival was calculated by using the observed survival in the patient cohort divided by the expected survival obtained from the general cancer-free population (can be identified from the nation-wide cancer registry), matched on age, sex, calendar period, county, and socioeconomic status. The expected survival was calculated with the Ederer II method^13^. The standard error of the observed survival was estimated by Greenwood’s formula^14^. Patients diagnosed between 1991 and 2015 were included in the study. Relative survival in adult patients (>20 years), with and without SPC, was measured from the time of diagnosis until death, immigration or 2015, whichever came first. Multivariable Cox proportional hazard regression model adjusting for sex, age at and calendar year of first cancer diagnosis and socioeconomic status was applied to assess hazard ratios (HRs) among patients with SPC in different prognostic groups compared to patients without SPC. In this model, the diagnosis of SPC was treated as a time-dependent variable in order to avoid the immortal time bias^15^. Trend test was performed by considering patients without any SPC, with SPC of good, moderate, and poor prognosis as continuous variable. All statistical analyses were performed in SAS (version 9.4) and R software. The study was approved by the ethical committee of the University of Lund.

Among 9338 CLL patients, a total of 1571 were diagnosed with SPC (16.8%) after a median (interquartile, 1–7) follow-up time of 4 years; 5639 deaths were recorded and of these 1122 (19.9%) were in patients diagnosed with a SPC. Among 718 HCL patients, a total of 119 were diagnosed with SPC (16.6%) after a median (interquartile, 2–11) follow-up time of 7 years; of 234 HCL deaths, 57 (24.4%) were recorded in patients with SPC. For CLL patients with second cancer of poor prognosis, the two main SPCs were lung and brain cancers, for those with moderate prognosis, they were NHL and colorectal cancer, and for those with good prognosis, they were skin (squamous cell) and prostate cancers. In HCL patients with second cancer, the two main SPCs were the same as with CLL in three groups of different prognosis. Corresponding case numbers, relative survival and 95% confidence intervals (CIs) for CLL are detailed in Supplementary Table 1.

Figure 1 shows relative survival for CLL and HCL, with and without SPC, and in patients with SPC in the three prognostic groups. For CLL, survival was significantly better (non-overlapping 95%CIs) for patients with SPC compared to those without SPC in the first year and years 2–5 after diagnosis (Fig. 1a, and Supplementary Table 1). The survival rate was reversed in subsequent years but was not significant. For HCL, the data between patients with and without SPC were essentially similar: in year 1, patients with SPC had significantly better survival than those without SPC but survival was reversed at subsequent periods, yet the differences were not significant (Fig. 1b, Supplementary Table 1). CLL patients in the good prognostic group showed excellent survival during the first years but with time the rate equalized with that of moderate prognosis (Fig. 1c). In the poor prognostic group, survival was lower at all follow-up times and the rates differed significantly from patients without SPC in follow-up times after year 1. For HCL the survival of good and moderate prognosis patients did not differ but those for poor prognosis were modestly suppressed (significant for years 7–16 years compared to patients without SPC).

**Fig. 1:**
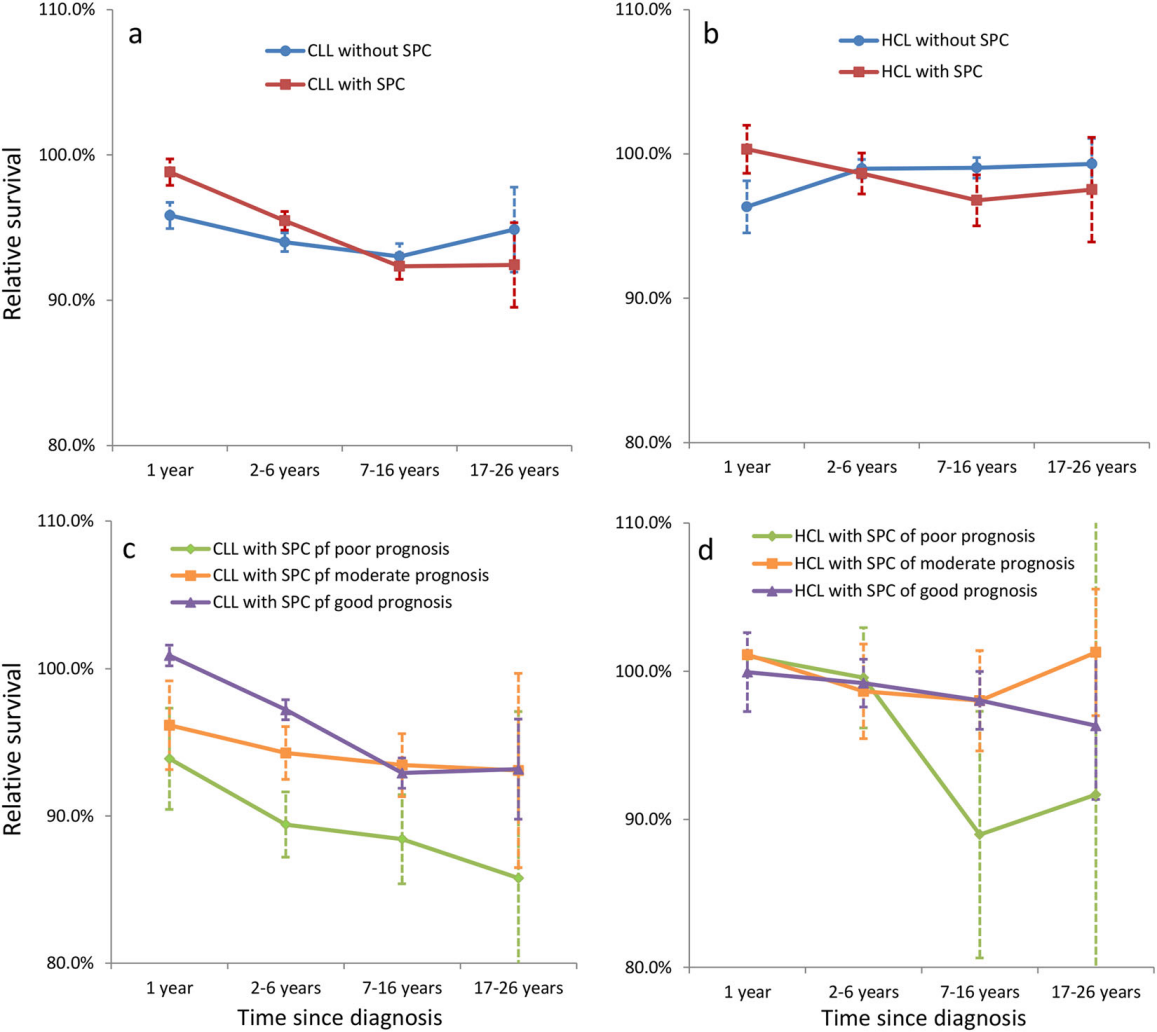
Relative survival in CLL and HCL patients according to diagnosis of SPC. Relative survival stratified by period (with 95% confidence interval) are shown for patients with and without SPC (**a** for CLL and **b** for HCL) as well as for patients with SPC of the good, morderate and poor prognosis (**c** for CLL and **d** for HCL). *SPC* second primary cancer, *CLL* chronic lymphocytic leukemia, *HCL* hairy cell leukemia

Patients had to survive some time to be diagnosed with SPC, which is a condition for immortality bias. To better understand the effect of different prognostic groups on the survival in SPC, a time-dependent analysis was necessary to avoid the bias. Multivariable Cox proportional hazard regression was performed by treating SPC diagnosis as time-dependent variable (Table 1). CLL patients with diagnosis of SPC of good prognosis experienced worse survival compared to those without any SPC diagnosis (HR = 1.76, 95%CI: 1.61–1.92). Patients with SPC of moderate (HR = 2.18, 1.76–2.70) and poor prognosis (HR = 5.83, 4.83–7.03) survived even worse. The trend test for HRs was highly significant (P-trend = 2 × 10^–16^). For HCL, the HRs for patients with SPCs of good, moderate, and poor prognosis were, respectively, 1.69 (1.11–2.57), 2.15 (0.92, 5.02), and 13.34 (4.92–36.33) and the trends were also significant (P-trend = 5 × 10^−6^).

**Table 1:** Hazard ratio in patients diagnosed with SPC of good, moderate and poor prognosis compared to those without SPC

Years since diagnosis	SPC of good prognosis		SPC of moderate prognosis		SPC of poor prognosis		P-trend
	*N*	HR (95% CI)	*N*	HR (95% CI)	*N*	HR (95% CI)	
CLL	681	1.76 (1.61–1.92)	177	2.18 (1.76–2.70)	220	5.83 (4.83–7.03)	2 × 10^−16^
HCL	31	1.69 (1.11–2.57)	11	2.15 (0.92–5.02)	9	13.34 (4.92–36.33)	5 × 10^−6^

Diagnosis of second cancer of unknown primary was not considered in any prognostic groups

The data shows that even for cancers with relatively good overall survival, those with SPC are a subgroup for whom survival may essentially deviate from patients without SPC and who may often be forgotten in prognostic evaluations. SPCs are a challenging issue concerning cancer survival and attempts to increase patient outcome cannot disregard the effect of SPCs. We tested, for the first time, the hypothesis that survival in SPCs would follow the survival experience known for first primary cancers. The hypothesis appeared to be correct and the trend tests between prognostic groups were highly significant, especially for CLL with large case numbers. Patients with SPC presented good survival in the early stage of follow-up time which is known as immortal time but experienced poor survival after diagnosis of SPC. This pattern of survival may indicate that some active drugs have led to better outcomes early but also caused mutations that subsequently lead to second malignancies.

Early mortality in CLL and HCL may be caused by severe infections. If the patient dies, SPCs may remain underreported. Another reason for underreporting of SPCs could be less vigilant diagnostic procedures in ill or frail patients^16^. Such underreporting may be a complication in survival studies thus masking the influence of SPCs but can be detected in the analysis of follow-up trends.

## Supplementary information

Supplementary Table 1
