# Supplementary material for: Types of second primary cancers influence survival in chronic lymphocytic and hairy cell leukemia patients
Source: Blood Cancer J. 2019 Mar 26;9(4):40. doi: 10.1038/s41408-019-0201-0 (PMC6435725; doi:10.1038/s41408-019-0201-0)
Supplement: Supplementary file 1 — Supplementary Table 1 [file 41408_2019_201_MOESM1_ESM.docx]

**Supplementary table 1** Relative survival in different follow-up time after diagnosis of CLL

| **Groups** | **1 year** | | | **2-6 years** | | | **7-16 years** | | | **17-26 years** | | |
| --- | --- | --- | --- | --- | --- | --- | --- | --- | --- | --- | --- | --- |
|  | N0 | N1 | **Relative survival (95% CI)** | N0 | N1 | **Relative survival (95% CI)** | N0 | N1 | **Relative survival (95%CI)** | N0 | N1 | **Relative survival (95% CI)** |
| Without SPC | 7767 | 557 | 95.8 (95.2-96.4) | 6973 | 2320 | 94.0 (93.6-94.3) | 3153 | 1532 | 93.0 (92.5-93.5) | 342 | 108 | 94.9(93.1-96.6) |
| With SPC | 1571 | 52 | 98.8 (97.9-99.7) | 1511 | 460 | 95.5 (94.8-96.1) | 952 | 546 | 92.3 (91.4-93.2) | 145 | 64 | 92.4 (89.5-95.3) |
| Poor prognosis | 256 | 21 | 93.9 (90.4-97.3) | 234 | 115 | 89.4 (87.2-91.6) | 114 | 77 | 88.4 (85.4-91.4) | 13 | 7 | 85.8(74.5-97.1) |
| Moderate prognosis | 241 | 14 | 96.1 (93.1-99.2) | 225 | 77 | 94.3 (92.5-96.1) | 132 | 74 | 93.4 (91.3-95.6) | 28 | 12 | 93.1(86.5-99.7) |
| Good prognosis | 1027 | 13 | 100.9 (100.2-101.6) | 1009 | 245 | 97.2 (96.5-97.9) | 688 | 379 | 92.9 (91.9-93.9) | 103 | 44 | 93.2(89.8-96.6) |

Diagnosis of second cancer of unknown primary was not considered in any prognostic groups.

N0, number of individuals alive in the initial follow-up of the time period; N1, number of individuals who died in the end of follow-up of the time period

CLL, Chronic lymphocytic leukemia, SPC, second primary cancer, 95%CI, 95% confidence interval
